# Virtual Physical Prehabilitation in Lung Transplant Candidates: A Proof-of-Concept Study

**DOI:** 10.3389/ti.2024.12355

**Published:** 2024-02-16

**Authors:** Nicholas Bourgeois, Larry C. Lands, Karina Prévost, Charles Poirier, Tania Janaudis-Ferreira

**Affiliations:** ^1^ Lung Transplant Program, Centre Hospitalier de l’Université de Montréal, Montreal, QC, Canada; ^2^ School of Physical and Occupational Therapy, McGill University, Montreal, QC, Canada; ^3^ Canadian Donation and Transplantation Research Program, Edmonton, AB, Canada; ^4^ Department of Pediatrics, Montreal Children’s Hospital-McGill University Health Centre, Montreal, QC, Canada; ^5^ Respiratory Epidemiology and Clinical Research Unit, Centre for Outcomes Research and Evaluation, Research Institute of the McGill University Health Centre, Montreal, QC, Canada

**Keywords:** lung transplantation, prehabilitation, telerehabilitation, exercise, rehabilitation

## Abstract

This study aimed to preliminary test the effectiveness of 12-week virtual physical prehabilitation program followed by a maintenance phase. The main objective was to estimate the extent to which it affects exercise capacity, frailty, lower limb strength and health-related quality of life (HRQOL) in lung transplant candidates. The program offered supervised strengthening exercises, independent aerobic exercises and weekly phone calls (maintenance phase). Primary outcome was the six-minute walk distance (6MWD). Secondary outcomes: the Short Physical Performance Battery (SPPB), five-times sit-to-stand test (5STS), the St George’s Respiratory Questionnaire (SGRQ) for HRQOL. Twenty patients were included (mean age 57.9; 6 women/14 men); fourteen completed the prehabilitation program and 5 completed the maintenance phase. There was no statistically significant improvement in 6MWD, SPPB or SGRQ after the 12-week program. Most patients either maintained or improved the 6MWT and SPPB scores. There was a significant improvement in the 5STS. After the maintenance phase, most patients either improved or maintained their scores in all outcomes except for the sub-score of symptoms in the SGRQ. A 12-week virtual physical prehabilitation program with a 12-week maintenance phase can help lung transplant candidates improve or maintain their physical function while waiting for transplantation.

## Graphical Abstract

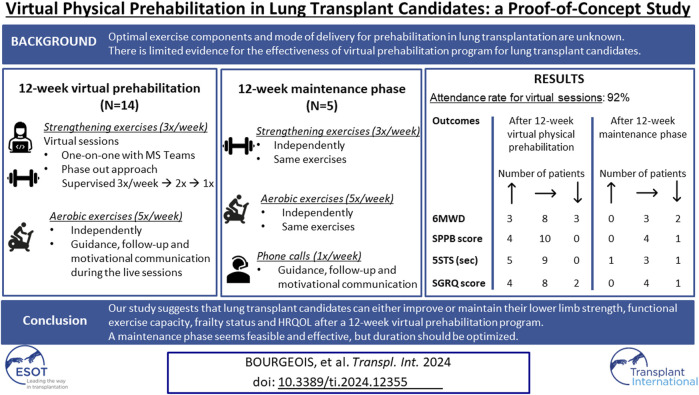

## Introduction

Individuals with advanced lung disease, including lung transplant candidates, present symptoms of dyspnea, decreased exercise capacity and muscle strength and are commonly frail; all of which impact their daily activities and societal roles [1, 2]. Limitations in exercise capacity in these individuals can negatively impact their clinical outcomes prior to and after lung transplantation [3]. For example, functional exercise capacity [assessed using the 6-min walk test (6MWT)] has been associated with mortality in patients awaiting lung transplantation [3] and following lung transplantation [3]. Frailty is also an important clinical factor as it has been shown to be associated with greater disability and delisting pre-lung transplant [4].

Although prehabilitation is recommended for lung transplant candidates to improve their physical and psychological health prior to the surgery and to obtain a faster recovery post-transplant [5], there is a very limited number of randomized controlled trials of exercise interventions in lung transplant candidates [6–8] and thus, the evidence is still scarce [5, 9]. A recent consensus statement on prehabilitation for solid organ transplantation candidates [9] stated that the optimal exercise components and mode of delivery for prehabilitation in lung transplantation are unknown.

Prehabilitation interventions included in the published literature are center-based [6, 10–13] or a mix of center-based with home-based [14–17]. Center-based programs may not be the optimal mode of delivery as transplant candidates may be waiting for their transplant in a distant location from the transplant centres. Unsupervised home-based exercises are feasible but may affect patients’ engagement and adherence [18, 19]. Technology tools incorporated into home-based delivery models have the potential to enhance uptake, adherence and communication between patients and providers as well as improve the efficiency for patient monitoring for safety and effectiveness [20]. During COVID-19 pandemic, many programs switched from center-based to virtual rehabilitation and continue to use this mode of delivery [21]. However, there is limited evidence for the effectiveness of virtual prehabilitation program for lung transplant candidates. Layton et al. [22] performed a 12-week home-based rehabilitation via an app in lung transplant candidates, but only patients with cystic fibrosis were included. Singer et al. [19] performed an 8-week home-based intervention through a mobile health application targeting frail lung transplant candidates, however, the intervention was mostly unsupervised, and the authors excluded patients with pulmonary hypertension.

In 2020, our team completed a retrospective study which examined the changes in functional exercise capacity in lung transplant candidates who had received counselling to perform exercises at home with no supervision [18]. This study demonstrated that the majority of the lung transplant candidates who performed the exercises at home were able to either increase or maintain their 6MWD during the waiting list period [18]. Due to its retrospective nature and the limited outcome measures included, a formal prospective evaluation of such program is required. Following the ORBIT model for Developing Behavioral Treatments for Chronic Diseases [23], our first prospective evaluation will be a proof-of-concept study which will determine if our improved intervention deserves more rigorous and costly testing using a randomized controlled trial.

The aim of this study is to preliminary test the effectiveness of an improved home-based exercise program with supervision and use of technology. The specific objectives are 1) to estimate the extent to which a 12-week virtual physical prehabilitation program affect exercise capacity, frailty, functional leg strength and health-related quality of life (HRQOL) in lung transplant candidates; 2) to estimate the extent to which any improvement in outcomes is maintained after a 12-week maintenance phase; 3) to assess the safety and acceptability of the improved intervention.

## Patients and Methods

This was a prospective longitudinal sequential study with three times points. The reporting of the findings is based on the CONSORT checklist extension for feasibility trials [24] and the Consensus on Exercise Reporting Template (CERT) [25]. The study was conducted at the Centre Hospitalier de l’Université de Montréal (CHUM) (Montreal, Quebec, Canada) between November 2021 and February 2023. The study was approved by the University of Montreal Health Centre Research Ethics Board.

### Participants

We recruited consecutive men or women (aged ≥18 years) who were being assessed to be listed for lung transplantation at the CHUM. Participants had to speak English or French and technologically capable of connecting (either independently or through household members) with an online videoconferencing platform. A tablet was lent to participants who did not have one. We excluded patients who were: 1) planning to be listed on the emergency waiting list as they would very likely not complete our intervention, 2) participating in a structured exercise program (hospital-based or home-based) and 3) hospitalized for any reason during the assessment for eligibility or waiting for the lung transplant. We also excluded patients who had pre-existing or newly identified cardiac, musculoskeletal, or neurological condition that could affect their exercise performance or otherwise render prehabilitation participation unsafe and patients who had pre-existing or newly identified significant cognitive impairment. The recruitment was made by the physiotherapist from the Lung Transplant Program. Medical clearance was given by a respirologist. Participants did not receive remuneration for this study other than being allowed to keep the fitness tracker used for the study.

### Intervention

The intervention was delivered by Willkin, an incorporated company that offers specialized kinesiologist services. The intervention consisted of a 12-week virtual physical prehabilitation program (induction phase) and a 12-week maintenance phase with independent home exercises.

The exercise program during the induction phase included lower and upper body strengthening (3 times/week) as well as independent aerobic exercises (5 times/week). The strengthening exercises consisted of functional exercises for lower extremities and weight exercises for upper extremities with existing home equipment (e.g., dumbbells, elastics or bottles/cans). No exercise equipment was given to patients. The strengthening exercises were administered through a screen interface over the Microsoft Teams video conferencing platform and lasted around 30 min. All live video sessions were performed in a one-on-one manner and sessions were not recorded.

The supervised sessions followed a phase-out approach to encourage progressive autonomy and long-term adherence to prescribed exercises. There were three supervised live video sessions/week during weeks 1–4; two supervised live video sessions/week during weeks 5–8 (and one independent session/week) and one supervised live video session/week during weeks 9–12 (and two independent sessions/week). The target intensity for strengthening exercises was a moderate intensity (rating of 3-4) on the Borg 0–10 scale [26] for dyspnea though initial intensities varied by patient. Training progression was tailored to each patient and were accomplished by a combination of repetition and/or set increases and by prescribing increasingly difficult exercises. The modifications were guided by participant feedback with the Borg scale improvements at the beginning and end of each session. Supplemental oxygen was titrated based on the initial 6MWT and patients were using the prescribed oxygen when doing the exercises. The exercise session was stopped if saturation dropped below 85%. Participants received a pulse oximeter if they did not have one.

Guidance and motivational communication were offered during the live sessions to encourage participants to perform the independent aerobic exercises. Recommendations were for at least 30 min of exercise 5 times per week, which could be done with a treadmill or stationary bike if available at home or walking in a mall or outdoors. A moderate-intense level with maintaining 3-4 in the Borg 0–10 scale [26] was recommended. As a safety measure, each participant wore a pulse oximeter for point-of-care heart rate and oxygen saturation information at each supervised or independent session.

After the 12-week virtual physical prehabilitation phase, patients were encouraged to maintain their exercise program that were prescribed previously (aerobic and strengthening) independently for 12 weeks. During this maintenance phase, they received weekly phone calls from the kinesiologist with motivational messages to keep them engaged. If patients were not transplanted within the 24-week period of the intervention, the physiotherapist of the Lung Transplant Program continued the follow-up according to the current standard of practice.

### Outcome Measures

Participants were assessed by the physiotherapist of the Lung Transplant Program. The outcomes were collected before (T0) and after the 12-week virtual physical prehabilitation phase (T1) and at the end of the maintenance phase (T2) (except for the acceptability outcome which was assessed at T2 only).

### Descriptive Measures

We collected age, sex, body mass index, primary pulmonary diagnosis, oxygen requirements, comorbidities, lung and cardiac function.

### Primary Outcome Measure

The primary outcome was functional exercise capacity (distance in meters) measured using the 6MWT according to the American Thoracic Society guidelines [27] for directives and encouragement. The predicted value of the 6MWT was calculated using the formula from normative data of healthy Canadians aged 45–85 years: 6MWD = 970.7 + (−5.5 × age) + (56.3 × gender), where females = 0, males = 1 [28].

Oxygen saturation and dyspnea [measured using the BORG scale 0–10 (26)] was assessed before, during and immediately after the 6MWT. The number of rests during the test was recorded. Oxygen requirement during the test was recorded as flow rate and delivery system and then converted to the estimated fraction of inspired oxygen (FiO_2_) using a suggested conversion table [29].

### Secondary Outcome Measures

Physical frailty was measured using the Short Physical Performance Battery (SPPB) [30]. The SPPB measures lower extremity function and is considered as a surrogate measure of physical frailty in adult lung transplant candidates [4, 31]. It has been found to have similar construct validity to the Fried Frailty Phenotype Index [31]. In lung transplant candidates, the SPPB has been categorized as frail (≤7/12), pre-frail [8, 9], and non-frail ≥10 [4]. The SPPB consists of three sub-tests scored from 0–4: standing balance, 4-m gait speed test and 5-repetition sit-to-stand (5STS) [32]. A score of 4 indicates the highest level of performance and 0 indicates inability to complete the task [32]. The results of the five-times sit-to-stand (5STS) component were also presented separately as a measure of functional lower limb strength [33].

Health-related quality of life (HRQOL) was assessed using the St George’s Respiratory Questionnaire (SGRQ). The SGRQ measures disease impact on overall health, daily life, and perceived wellbeing in individuals with chronic lung diseases including lung transplant candidates [34]. Adherence was used using a multi-modal strategy as suggested by the World Health Organization [35]. Adherence to the exercise program was monitored using two diaries and a fitness tracker (AK1980, China) which was used as a pedometer to record steps. To reach the objectives of 30 min of exercise per day, we used a proposed calculation of 3000 steps for 30 min of walking [36] as a decision for adherence. Participants recorded their number of steps daily in a document and recorded their unsupervised exercise sessions in another document including the duration of aerobic training, type of strengthening exercises including number of sets and repetitions and series. Both documents were in paper format and were retrieved at the end of the study, The acceptability of the intervention was assessed using a semantic differential scale that consisted of 16 questions graded on a 7-point Likert scale with a total possible score of 48. For analysis, answers with grades 1 to 3 were classified as being in agreement with the statement, 0 classified as neutral and −1 to −3 as being in disagreement. Adverse outcome, costs in Canadian dollars of therapist hours and equipment were documented.

### Analysis

Based on the data from our retrospective study [18], we required a sample size of 5 to achieve a power of 80% and a level of significance of 5% (two sided) for detecting a mean difference of 85.8 m in the 6MWT between pre- and post-intervention, assuming that the SD of the difference is 42.8 m (the minimal clinically important difference (MCID) in this population is 30 m [37]). However, to be powered for our secondary outcome (frailty measured by the SPPB), 11 patients were required (based on data from Wickerson et al. [4]; mean difference of 1 point and standard deviation of 1). To account for a 15% refusal rate [38] and loss to follow-up (patients who would eventually be transplanted before the end of the intervention), we planned to include 20 patients. Normality of the data was tested with the Shapiro-Wilk Test. Paired t-tests were performed to examine the changes in the outcomes pre (T0) vs post induction phase (T1) in normally distributed data. Wilcoxon rank test was used if the normality of the data distribution was not obtained. Due to the small number of participants completing the maintenance phase, the data on the difference between the end of the induction phase (T1) and the end of maintenance phase (T2) were reported descriptively. All *p*-values are two-tailed, and values < 0.05 were considered statistically significant. We calculated the effect size (ES) of each outcome using the Cohen’s d calculation and degree of ES [39]. We analyzed the changes in each outcome related to the MCID of each of them. We used the following MCID: 30m for the 6MWD (37), 1.7 s for the 5STS [40], 8 for the SGRQ [41, 42] and 1 for the SPPB [43]. Statistical analysis was performed using SPSS software (SPSS, version 26.0; IBM).

## Results

### Participant Characteristics

**FIGURE 1:**
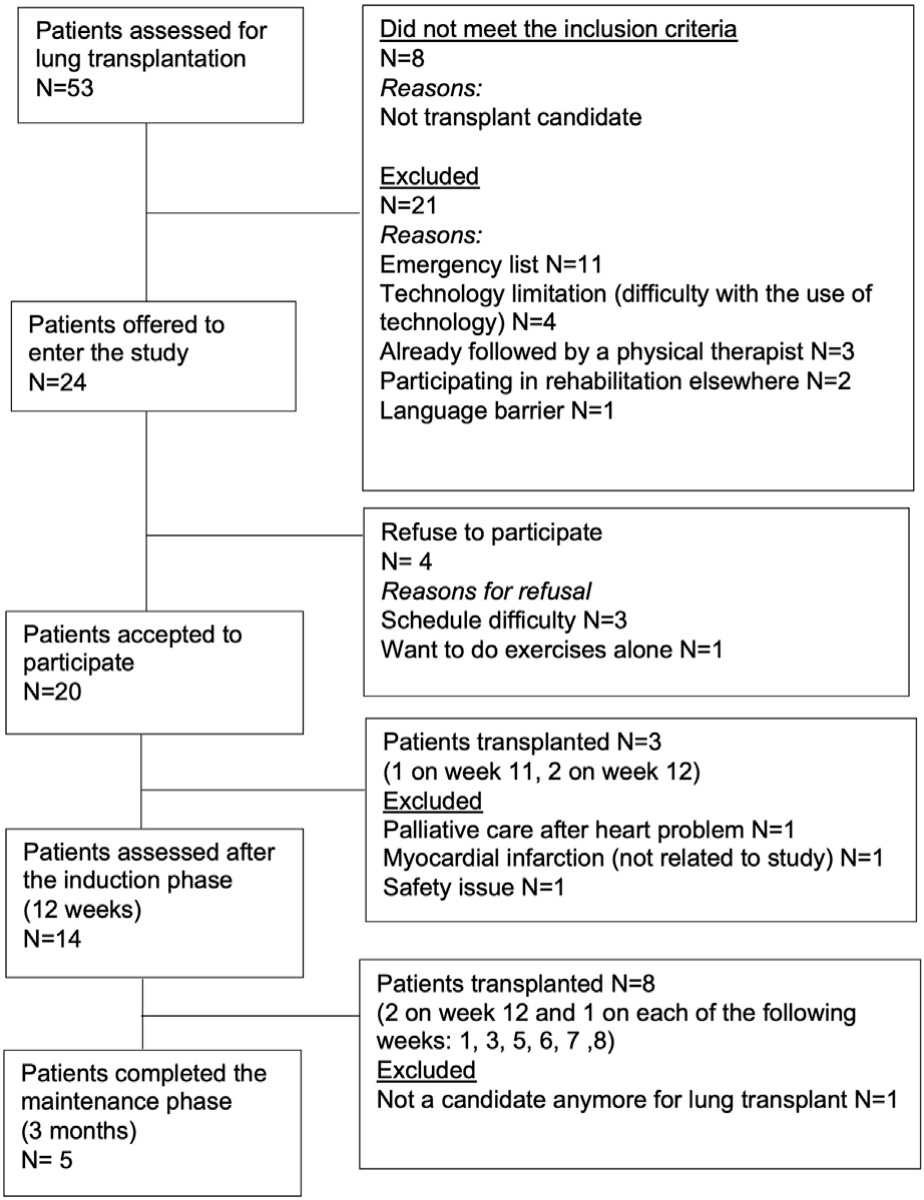
Flow-chart.

Fifty-three patients referred for lung transplantation between November 2021 and August 2022 were assessed for eligibility. After applying the inclusion and exclusion criteria, twenty-four patients (45%) were offered to enter the study and 20 accepted to participate. Fourteen patients completed the induction phase (70% and five completed the maintenance phase (25%) (Figure 1). During the virtual prehabilitation phase, three patients were transplanted before the reassessment and three patients were excluded for medical reasons (Figure 1). Of the 14 participants who started the maintenance phase, 8 were transplanted before the final assessment and one was excluded as he was no longer a candidate for transplant. Baseline characteristics of the 20 patients are presented in the Table 1. When comparing the 14 patients who completed the virtual prehabilitation phase with the 6 patients who did not complete it, no statistically significant differences in their baseline characteristics were found.

**TABLE 1:** Participant characteristics at baseline.

	*Patients included (N = 20)*
Age (years)	57.9 ± 11.0
Sex: Female/Male [n (%)]	6 (30)/14 (70)
Primary diagnosis [n (%)]	
ILD	9 (45)
COPD	6 (30)
CF	2 (10)
Retransplant	1 (5)
PAH	1 (5)
Sclerodermia	1 (5)
Comorbidities (>1 patient) [n (%)]	
Gastroesophageal reflux disease	6 (30)
Hypertension	4 (20)
Osteoporosis	4 (20)
Dyslipidemia	4 (20)
Anxiety	4 (20)
Coronary heart disease	2 (10)
Diabetes	2 (10)
Anemia	2 (10)
*Clinical characteristics*	
BMI (kg/m^2^)	23.2 ± 4.4
Home oxygen at rest (% FiO_2_)	23.7 ± 4.4
Home oxygen at exercise (% FiO_2_)	31.1 ± 10.3
FEV1 (% pred)	43.0 ± 22.0
FVC (% pred)	52.6 ± 12.9
DLCO (% pred)	65.5 ± 27.0
LVEF (%)	54.5 ± 10.7
PAP (mmHg)	54.4 ± 30.3
*Outcome measures at baseline*	
6MWT	
6MWD (m)	342.7 ± 70.0
Percentage predicted 6MWD (%)	50.2 ± 12.3
Borg max (/10)	5.8 ± 1.4
HR max (bpm)	112.8 ± 15.8
FiO_2_ during test (%)	33.5 ± 9.9
SPPB	
Total score (/12)	11.4 ± 0.9
Balance score (/4)	4.0 ± 0.2
4MGS score (/4)	3.9 ± 0.4
5STS score (/4)	3.6 ± 0.7
5STS (sec)	10.3 ± 2.3
SGRQ	
Symptoms score (/100)	61.3 ± 19.8
Activities score (/100)	82.1 ± 13.0
Impacts score (/100)	56.3 ± 21.6
Total score (/100)	65.1 ± 17.2

Values are [mean ± SD] if not mentioned otherwise.

Abbreviations: ILD, interstitial lung diseases; COPD, chronic obstructive lung disease; CF, cystic fibrosis; PAH, pulmonary arterial hypertension; BMI, Body-Mass Index; FiO2, fraction inspired oxygen; FEV1, forced expiratory volume in 1 s; FVC, forced vital capacity; DLCO, diffusing capacity of the lungs for carbon monoxide; LVEF, left ventricular ejection fraction; PAP, pulmonary arterial pressure; 6MWT, six-minute walk test; 6MWD, six-minute walk distance; HR, heart rate; SPPB, short physical performance battery; 5STS, five time sit-to-stand; 4MGS, 4-m gait speed; SGRQ, St George’s Respiratory Questionnaire.

### Changes After the 12-week Virtual Physical Prehabilitation

**FIGURE 2:**
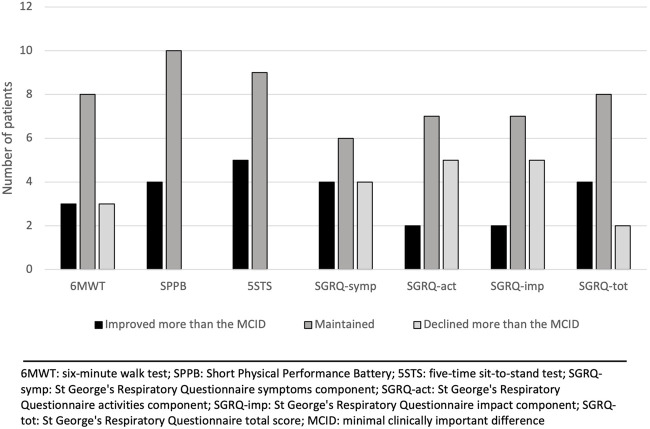
Changes in outcomes after the 12-week virtual physical prehabilitation phase relative to the minimal clinically important difference (MCID) of each outcome.

Changes in outcomes after the 12-week virtual physical prehabilitation phase are presented in Table 2. No statistically significant difference was noted in the 6MWT, in distance in meters or % of predicted distance. There was no statistically significant difference in the Borg scale level at the end of the walking test but there was statistically significant increase in the FiO_2_ used during the 6MWT (mean change of 7.1%, *p* = 0.012, ES = 0.53) (The decision to increase the FiO_2_ during the 6MWT was made by the clinical team). We found a statistically significant decrease in the 5STS test (mean change of 1.4 s, *p* = 0.009, ES = 0.61). There was no statistically significant difference in the SPPB score (*p* = 0.059) or in the SGRQ total score and the 3 sub-scores. When we examine the effect size, we can see a trend in improvement in the SPPB score, decrease in the Borg scale during the 6MWT as well as improvement in the impact sub-score of the SGRQ. When the changes in the 6MWT was compared to its MCID, we noted that 11 patients either improved or maintained their 6MWT scores. As for the other outcomes, the majority of the patients improved or maintained their scores in all the outcomes (Figure 2).

**TABLE 2:** Changes in outcomes after the 12-week virtual physical prehabilitation.

	*Baseline (T0) (N = 14)*	*Post 12* *weeks (T1) (N = 14)*	*Mean change*	*p-value*	*Effect size*
6MWT					
6MWD (m)	357.4 [315.6–399.1]	359.6 [305.7–413.5]	2.21 [−25.7–21.2]	.842	0.03
	*356 (254*–*464)*	*359 (192*–*528)*			
% predicted 6MWD	53.0 [45.9–60.1]	53.5 [44.6–62.4]	0.5 [−4.1–3.1]	.770	0.04
	*52.5 (36.0*–*75.0)*	*49.0 (27.0*–*78.0)*			
Borg max (/10)	5.6 [4.7–6.4]	5.0 [3.9–6.1]	-0.6 [−1.9–0.7]	.358	0.36
	*6 (3*–*8)*	*4 (2*–*9)*			
HR max (bpm)	111.5 [102.7–120.3]	110.5 [103.0–118.0]	−1.0 [−4.5–2.5]	.549	0.07
	*114 (82*–*129)*	*110 (81*–*128)*			
% FiO_2_ during test^#^	33.6 [27.9–39.4]	40.8 [31.2–50.4]	7.1 [1.9–12.4]	.012*	0.53
	*30 (21*–*55)*	*36 (21*–*75)*			
SPPB score					
Total score^#^	11.4 [10.8–12.0]	11.8 [11.5–12.0]	0.4 [−0.1–0.9]	.059	0.56
	*12 (9*–*12)*	*12 (11*–*12)*			
Balance score^#^	3.9 [3.8–4.0]	4.0 [4.0–4.0]	0.1 [−0.1–0.2]	.320	0.52
	*4 (3-4)*	*4 (4*-*4)*			
4MGS score^#^	3.9 [3.6–4.0]	3.9 [3.8–4.0]	0.1 [−0.1–0.2]	.317	0.22
	*4 (3*-*4)*	*4 (3*-*4)*			
5STS score^#^	3.6 [3.1–4.0]	3.9 [3.6–4.0]	0.3 [−0.1–0.6]	.102	0.52
	*4 (2*–*4)*	*4 (3-4)*			
5STS (sec) ^#^	10.0 [8.5–11.5]	8.6 [7.5–9.8]	−1.4 [−2.3–−0.5]	.009*	0.61
	*9.3 (7.2*–*12.1)*	*8.1 (6.2*–*13.2)*			
SGRQ					
Symptoms Score	56.4 [45.3–67.4]	53.5 [40.1–66.9]	−2.8 [−11.0–5.3]	.465	0.13
	*57.6 (26.2*–*83.8)*	*53.4 (0*–*92.8)*			
Activities score^#^	81.4 [74.2–88.5]	83.9 [75.4–92.3]	2.5 [−6.6–11.6]	.314	0.19
	*79.5 (53.4*–*100)*	*89.2 (41.6*–*100)*			
Impacts score	52.3 [39.6–65.1]	46.3 [36.2–56.4]	−6.0 [−14.4–2.3]	.144	0.30
	*55.9 (11.7*–*80.5)*	*53.9 (16.1*–*70.2)*			
Total score	62.0 [52.1–71.9]	59.1 [50.2–67.9]	−2.9 [−10.2–4.4]	.401	0.18
	*65 (30.7*–*84.5)*	*65 (26.2*–*76.1)*			

Paired samples *t*-test were used for normally distributed data.

Wilcoxon rank test was used for non-normally distributed data (see # above).

Mean [95% CI].

Median (Min-Max).

*p < 0.05.

Effect size was calculated with Cohen d.

Abbreviations: 6MWT, six-minute walk test; 6MWD, six-minute walk distance; HR, heart rate; FiO2, fraction inspired oxygen; SPPB, short physical performance battery; 5STS, five time sit-to-stand; 4MGS, 4-m gait speed; SGRQ, St George’s Respiratory Questionnaire.

### Changes After the Maintenance Phase

**FIGURE 3:**
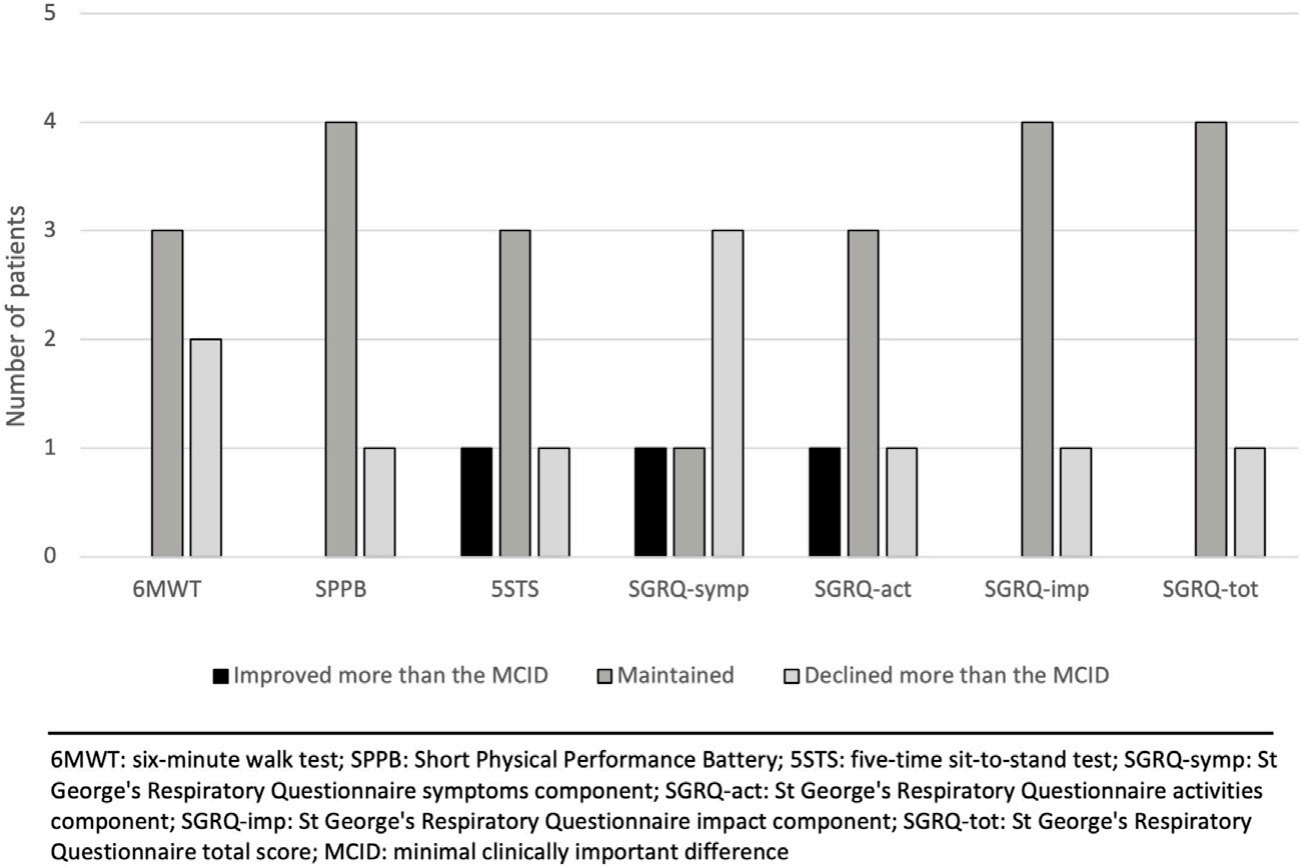
Changes in outcomes after the maintenance phase relative to the minimal clinically important difference (MCID) of each outcome.

Five patients completed the maintenance phase. Reasons for non-completion are shown in Figure 1. Changes in outcomes (compared to the 12-week prehabilitation follow-up) for each patient individually after the maintenance phase are presented in Table 3. We observed a mean decrease of 32.4 m in the 6MWD with an increase in the Borg scale and an increase in the FiO_2_. Compared with the MCID for each outcome, the majority of the patients improved or maintained their scores in all the outcomes except for the sub-score of symptoms in the SGRQ where 3 patients declined their score (Figure 3).

**TABLE 3:** Changes in outcomes in each patient after the 12-week maintenance phase.

	*6MWT*					*SPPB score*	*5STS (sec)*	*SGRQ*			
	*6MWD (m)*	*% pred 6MWD*	*Borg* max *(/10)*	*HR* max *(bpm)*	*% FiO*_*2*_ *during test*			*Symptoms Score*	*Activities score*	*Impacts score*	*Total score*
Patient 1											
T1	316	49	6	102	36	12	7.9	63.9	80.5	70.2	72.5
T2	306	48	7	93	51	12	6.6	45.4	73	66.7	65.4
*Change*	*−10*	*−1*	*1*	*−9*	*15*	*0*	*−1.34*	*−18.5*	*−7.5*	*−3.5*	*−7.1*
Patient 2											
T1	356	49	6	114	21	11	13.2	92.8	92.5	61.5	76.1
T2	351	48	5	106	28	10	16.5	86.4	92.5	64.4	76.6
*Change*	*−5*	*−1*	*−1*	*−8*	*7*	*−1*	*3.35*	*−6.4*	*0*	*2.9*	*0.5*
Patient 3											
T1	437	67	3	112	50	12	7.6	36.7	72.2	39.2	49.1
T2	372	57	6	106	75	12	5.9	56.3	92.5	46.6	62.1
*Change*	*−65*	*−10*	*3*	*−6*	*25*	*0*	*−1.7*	*19.6*	*20.3*	*7.4*	*13*
Patient 4											
T1	490	76	3	108	75	12	9.9	0	41.6	25.7	26.2
T2	462	71	3	113	75	12	9.3	11.1	17.3	24.1	20
*Change*	*−28*	*−5*	*0*	*5*	*0*	*0*	*−0.6*	*11.1*	*−24.3*	*−1.6*	*−6.2*
Patient 5											
T1	362	58	5	125	44	12	7.6	53.4	92.5	55.1	66.5
T2	308	49	7	127	44	12	8.8	68.4	92.5	49.6	65.7
*Change*	*−54*	*−9*	*2*	*2*	*0*	*0*	*1.2*	*15*	*0*	*−5.5*	*−0.8*
All											
T1 mean	392.2	59.8	4.6	112.2	45.2	11.8	9.2	49.4	75.9	50.3	58.1
T1 SD	70.0	11.7	1.5	8.5	19.9	0.4	2.4	34.3	21.0	17.8	20.6
T2 mean	359.8	54.6	5.6	109.0	54.6	11.6	9.4	53.5	73.6	50.2	58.0
T2 SD	63.7	9.9	1.7	12.4	20.4	0.9	4.2	28.1	32.6	17	21.9
*Mean Change*	−32.4	−5.2	1	−3.2	9.4	−0.2	0.2	4.2	−2.3	−0.1	−0.1

T1: value after 12-week virtual physical prehabilitation phase.

T2: value after maintenance phase.

Abbreviations: 6MWT, six-minute walk test; 6MWD, six-minute walk distance; HR, heart rate; FiO2, fraction inspired oxygen; SPPB, short physical performance battery; 5STS, five time sit-to-stand; SGRQ, St George’s Respiratory Questionnaire; SD, standard deviation.

### Adherence

The average attendance rate of the virtual sessions for the 14 participants who completed the 12-week virtual physical prehabilitation phase was 91.9% (range 75%–100%). Four patients completed the diary for the independent exercise sessions, three patients partially and 7 patients did not complete the diary. The main reason for not completing the diary was that they forgot to record their sessions or lost the diary. As for the daily steps diary, six patients completed it correctly, one patient partially and 7 patients did not complete it. Patients provided the same reasons for not completing the daily steps diary. The average steps per day ranged from 1,296 to 5,901 in the 8 patients that filled out the daily steps diary. Using the proposed cut-off of 3,000 steps, only 4 patients had adequate adherence to the aerobic exercises.

### Adverse Events

No adverse events were reported during the live training sessions, independent sessions at home or walking.

### Cost

In our study, two kinesiologists spent approximately 480 h to deliver the exercise program to the participants. At $40 Canadian dollars/hour, this represents a total budget of $19,200 Canadian dollars. As for the evaluation session by the physiotherapist, we calculated a total of 1.5 h per participant, for a total of 30 h. At a salary of $50 Canadian dollars/hour, this represents a total of $1,500 Canadian dollars. We bought for $3,500 worth of equipment: pulse oximeters, tablets, fitness trackers. We did not need to lend any tablets and only loaned just one oximeter. The overall cost of our intervention in 20 individuals was $24,200 Canadian dollars. This led to a cost per patient of $1,210 Canadian dollars.

### Acceptability

Seventeen participants completed the acceptability questionnaire. The average score of the acceptability questionnaire was high at 45.5 (range 33–48, SD 3.8; maximal score is 48). See Table 4 for more details on the questions and answers of the acceptability questionnaire.

**TABLE 4:** Responses of the acceptability questionnaire.

Questions from questionnaire	Agree with this sentence (% of participants)
To offer this exercise program for people awaiting lung transplantation is
• Good idea	17 (100%)
• Pleasing	17 (100%)
• Easy^a^	16 (94%)
• Helpful	17 (100%)
• Simple^b^	16 (94%)
My family and/or friends liked that I participated in the exercise program^a^	16 (94%)
The exercises provided in the program were relevant to me	17 (100%)
I see the need for this virtual home-based exercise program in my life	17 (100%)
I think I benefited from this exercise program	17 (100%)
I felt confident to perform all exercises without assistance	17 (100%)
It was easy to learn how to perform the exercises	17 (100%)
It was easy to connect with the physiotherapist via Teams^b^	16 (94%)
I would recommend this virtual home-based exercise program to others	17 (100%)
The length of the program was good	17 (100%)
The number of exercises was good	17 (100%)
I intend to continue to do the exercises even after the program has finished	17 (100%)

N = 17.

^a^ 1 participant was neutral for this item.

^b^ 1 participant disagree with this item.

## Discussion

This prospective longitudinal study demonstrated that a 12-week virtual prehabilitation program can improve lower limb strength as measured by the 5STS and maintain exercise capacity, frailty status and HRQOL in lung transplant candidates. A 12-week maintenance phase can either improve or maintain these outcomes. There was a high drop-out rate in the maintenance phase due mainly to intercurrent transplantation. The prehabilitation program was well accepted by patients and had a high attendance rate and no adverse events.

Although most of the participants were able to improve or maintain their 6MWD after the 12-week virtual physical prehabilitation phase, there was no statistically significant improvement in this outcome. Similarly, Singer et al. [19] did not demonstrate improvement in 6MWD after an unsupervised home-based exercise training delivered via a mobile device to frail lung transplant candidates. In addition, Layton et al. [22] did not find improvements in 6MWD after a telerehabilitation offered to lung transplant candidates with cystic fibrosis. In contrast, prospective studies that offered hospital-based prehabilitation programs to lung transplant candidates have shown significant improvements in the 6MWD after the period of prehabilitation [6, 11, 44, 45]. This discrepancy could be because the hospital-based programs were able to offer a more intense aerobic exercise with supervision. In our study, the aerobic component of the program was not supervised. Most of the patients did not complete the diary to record the number of aerobic exercise sessions, therefore, we are unable to determine whether patients adhered to this part of the program. We also observed that the amount of oxygen that participants required to perform the 6MWT after the intervention was higher than what it was required before intervention which reflects a higher hypoxemia and may represent a progression of the underlying disease [46, 47]. Considering the progressive nature of the end-stage lung disease, maintaining the functional walking capacity of transplant candidates during the waiting time is a good outcome.

There was no statistically significant change in the SPPB after the prehabilitation, however, the mean change was close to reach statistical significance (*p* = 0.059). This is in line with the findings by Singer et al. [19] which found no statistically significant difference in the SPPB after the prehabilitation program even though their patients had lower SPPB scores at baseline than our patients (mean of 9.7 vs. 11.4) [19]. Byrd et al. [45] showed a statistically significant improvement in the SPPB after a 1 month outpatient rehabilitation in lung transplant candidates and the effect size of their cohort was similar to our study (0.54 vs. 0.56). The high SPPB scores at baseline in our study is explained by the fact that patients with limited functional status and who are frail are normally not listed for transplantation in our centre. This aligns with the recent consensus document for the selection of lung transplant candidates where frailty is considered a risk factor and limited functional status as an absolute contraindication if there is no potential for rehabilitation [48]. However, perhaps the frail patients could be the ones to target with prehabilitation as they are the ones that would benefit the most so that they can be considered for transplantation.

We found statistically significant improvement in one component of the SPPB, the 5STS. As the virtually one-on-one sessions focused on strengthening exercises and attendance to these sessions was high, this result was expected. Wickerson et al. [4] showed in an hospital-based outpatient program an improvement in the 5STS component of the SPPB after 6 weeks of prehabilitation. Byrd et al. [45] also found an improvement in 5STS after 4 weeks of inpatient prehabilitation. As quadriceps strength has been associated with intensive care length of stay and exercise capacity [49], increasing lower limb strength may positively impact post-transplant outcomes.

There was no significant improvement in HRQOL in our study. The symptom and impact components of the SGRQ as well as the total score improved, but not enough to reach statistical significance. In lung transplant candidates, all domains of quality of life are affected to some degree but physical functioning appears to be more affected than mental health [50]. As the goal of exercise training is to improved physical function, which in fact we observed in our study, one would expect that the HRQOL in our participants would improve. However, a decline in HRQOL in transplant candidates can occur due to fatigue, loss of self-esteem, anxiety and depression related to the prognosis of end-stage disease which might outweigh the effect of exercise [51]. Some studies of exercise interventions in lung transplant candidates have shown improvements in HRQOL [11, 13, 15], others in some components only [12, 14, 16]. In contrast, Li et al. [10] have noted a decline in all component of the SGRQ. In addition, a systematic review of exercise interventions in solid organ transplantation has not shown improvement in HRQOL when comparing intervention with control groups [52]. Finally, the non-difference in the SGRQ might be because we were not powered to see a difference in this outcome.

There was a high number of patients who underwent transplant during our study and therefore did not complete the post-interventions assessments. Three patients were transplanted during the 12-week virtual physical prehabilitation phase and eight during the 12-week maintenance phase. The duration of the induction phase was informed by the findings of a systematic review on exercise in solid organ transplant candidates [52]. Studies that reported improvements in exercise capacity had an exercise program duration longer than 10 weeks [52]. Additionally, many studies in lung transplant prehabilitation have used a 12-week program [9, 52]. The loss of patients during the induction phase might have prevented us from seeing differences in some outcomes. Also, we were not able to see any difference and perform complete analysis of the maintenance phase as 8 of the remaining 14 patients after the induction phase were transplanted before the end of this phase. The intervention duration was designed based on experience without knowing the recent change in the waiting time. Indeed, the increasing number of lung transplants in our centre in the last few years significantly decreased the waiting time for transplant (from 12 months to 4 months in average).

In this study, we calculated that cost of our intervention per patient was $1,210 Canadian dollars for the healthcare system. As our study was not aimed to perform cost analysis, some expenses were not recorded thoroughly and a comparison between our standard care could not been done. However, a systematic review by Grigorovich et al. [53] on economic analysis of home-based telerehabilitation found that telerehabilitation may result in similar or lower costs as in-person rehabilitation. The social impact and expenses for patients should also be considered. During our intervention, patients could stay at home until the date of transplant as opposed to travelling or moving closer to a transplant or rehabilitation centre to perform the prehabilitation program.

One of the strengths of this study includes the one-on-one virtual strengthening sessions with the presence of a kinesiologist while other studies [19, 22] included applications with videos of exercises. The one-on-one sessions allowed direct supervision of the participants to adjust exercises and monitor vitals. As non-adherence to home-based rehabilitation can reach 50%, strategies to provide direct feedback, monitor symptoms and performance of exercises can improve self-efficacy and increase adherence to exercises as patient feel better supported [54]. Another strength is the inclusion of a maintenance phase after the induction period. As transplant date is not known, limiting an intervention to a certain amount of time could mean that the patients would have to maintain exercises for a longer period than 12 weeks. Interestingly, our recruitment rate was higher (83%) than those in Layton et al. (72%) and Singer et al. (65%). A possible explanation could be that our recruitment was made by the physiotherapist of the Lung Transplant Program. Since our study was conducted after the start of the COVID-19 pandemic, it may also be that patients and their families were more familiar with teleconferencing platforms at that time. Finally, we included participants with a wide range of diagnoses (ILD, CF, COPD, PAH, sclerodermia, and one patient for retransplantion).

There are several limitations to this study. First, our final sample size may have limited us from reaching statistically significance in our outcomes. The absence of a control group limited us from drawing a definitive conclusion on the effectiveness of the intervention. However, as the main goal of this study was to preliminary test our prehabilitation program following the ORBIT model, adding a control group in this phase was not recommended [23]. Another limitation was the uncertain adherence to the program. There was a larger number of diary non-completion for independent sessions which makes it difficult to know the exact frequency of the walking program, especially during the maintenance phase. Also, although we provided an exercise booklet and directives to the participants about the strengthening exercises, the number of sets and repetitions were not recorded. In future trials, the therapist should ask participants every week what they did for their independent sessions and record it with more details. During the maintenance phase, the same information can be asked during the weekly phone calls. Although the inclusion of a maintenance phase after the induction period was a strength of our study, 64% of the patients dropped out during this period, mainly because of intercurrent transplantation. The optimal duration for the maintenance phase should be determined in future trials. Finally, this study participants are not reflective of the entire lung transplant candidate’s population. We excluded patients that were on the emergency list for transplantation since they were more likely to be transplanted before the end of the induction phase or because a large proportion of them were hospitalized before the transplant.

## Conclusion

Our study suggests that lung transplant candidates can either improve or maintain their lower limb strength, functional exercise capacity, frailty status and HRQOL after a 12-week virtual prehabilitation program that is safe and acceptable to patients. Offering a maintenance phase seems feasible and effective, but optimal duration of this phase should match the transplant wait times of each center. Whether this prehabilitation program can impact post-transplant outcomes still needs further study.

## Data Availability

The raw data supporting the conclusion of this article will be made available by the authors, without undue reservation.
